# Bariatric surgery emphasizes biological sex differences in rodent hepatic lipid handling

**DOI:** 10.1186/s13293-017-0126-x

**Published:** 2017-01-28

**Authors:** Bernadette E. Grayson, Ruth Gutierrez-Aguilar, Joyce E. Sorrell, Emily K. Matter, Michelle R. Adams, Philip Howles, Rebekah Karns, Randy J. Seeley, Darleen A. Sandoval

**Affiliations:** 1 Department of Neurobiology and Anatomical Sciences, University of Mississippi Medical Center, Jackson, MS USA; 2 Divsion de Investigacion, Facultad de Medicina, Universidad Nacional Autónoma de México and Laboratorio de Enfermedades Metabólicas Obesidad y Diabetes, Hospital Infantil de México “Federico Gómez”, Mexico, Mexico; 3 Department of Internal Medicine, University of Cincinnati, Cincinnati, Ohio USA; 4 Cincinnati Children’s Hospital Medical Center, Cincinnati, Ohio USA; 5 Department of Pathology, University of Cincinnati, Cincinnati, Ohio USA; 6 Bioinformatics Core, Cincinnati Children’s Hospital and Medical Center, Cincinnati, Ohio USA; 7 Department of Surgery, University of Michigan, 2800 Plymouth Rd., Ann Arbor, MI 48109 USA

**Keywords:** Vertical sleeve gastrectomy, Triglycerides, Liver, Bariatric surgery, Sex difference

## Abstract

**Background:**

Eighty percent of patients who receive bariatric surgery are women, yet the majority of preclinical studies are in male rodents. Because sex differences drive hepatic gene expression and overall lipid metabolism, we sought to determine whether sex differences were also apparent in these endpoints in response to bariatric surgery.

**Methods:**

Two cohorts of age-matched virgin male and female Long-Evans rats were placed on a high fat diet for 3 weeks and then received either Sham or vertical sleeve gastrectomy (VSG), a surgery which resects 80% of the stomach with no intestinal rearrangement.

**Results:**

Each sex exhibited significantly decreased body weight due to a reduction in fat mass relative to Sham controls (*p* < 0.05). Microarray and follow-up qPCR on liver revealed striking sex differences in gene expression after VSG that reflected a down-regulation of hepatic lipid metabolism and an up-regulation of hepatic inflammatory pathways in females vs. males after VSG. While the males had a significant reduction in hepatic lipids after VSG, there was no reduction in females. *Ad lib*-fed and fasting circulating triglycerides, and postprandial chylomicron production were significantly lower in VSG relative to Sham animals of both sexes (*p* < 0.01). However, hepatic VLDL production, highest in sham-operated females, was significantly reduced by VSG in females but not males.

**Conclusions:**

Taken together, although both males and females lose weight and improve plasma lipids, there are large-scale sex differences in hepatic gene expression and consequently hepatic lipid metabolism after VSG.

**Electronic supplementary material:**

The online version of this article (doi:10.1186/s13293-017-0126-x) contains supplementary material, which is available to authorized users.

## Background

To date, bariatric surgical procedures are the most successful method to treat obesity and resolve metabolic comorbidities. Vertical sleeve gastrectomy (VSG) is a particular type of bariatric surgery which removes about 80% of the stomach along the greater curvature and involves no intestinal rearrangement. VSG is rapidly expanding in utilization due to the fact that it is highly efficacious at causing weight loss (60–65%) [1, 2] and improving type 2 diabetes and hyperlipidemia [3].

The mechanism(s) that underlie the efficacy of bariatric surgery are unknown but our contention is that there are key molecular events triggered by surgery that have lasting effects on metabolic homeostasis. Many groups, including our own, are focused on utilizing rodent models of bariatric surgery in order to identify these molecular events [4–7]. However, despite the fact that women represent approximately 80% of the bariatric surgery patient population, the vast majority of the preclinical work, has been in male rodents (see [8–10] as exceptions). Overall, this distinction may not seem important; surgery is highly efficacious regardless of sex. However, biological sex has potent effects on lipid metabolism that extends beyond region-specific fat distribution. For example, in response to metabolic stress, such as weight loss or exercise, fat is mobilized more readily in women [11–14]. This is also evident from an evolutionary perspective where transcriptional profiling of hepatic genes revealed that 70% of 1249 genes were upregulated in females and many of those genes were related to lipid metabolism [15]. Thus, if we are to understand the molecular changes underlying the success of surgery, we cannot ignore the potential influence of sex on the affected pathways.

Our previous data have demonstrated a *reduction* in circulating [16] and hepatic [17] triglycerides in male rats following VSG. In contrast, female rats that had VSG prior to pregnancy but were sacrificed several months after pregnancy and lactation, demonstrated *elevated* hepatic triglycerides [8]. It remains unknown whether this would also be seen in female rats that had never been pregnant. Thus, the purpose of the present study was to directly compare hepatic lipid metabolism in virgin male and female rats after VSG.

## Methods

### Animals

All procedures for animal use were approved by the University of Cincinnati Institutional Animal Care and Use Committee and follow the guidelines outlined in the National Institutes of Health guide for the care and use of laboratory animals (NIH Publications No. 8023, revised 1978). Two cohorts of age-matched (8 weeks) Long Evans rats (male body weight 225–250 g, female body weight 175–200 g) (Harlan Laboratories, Indianapolis, IN) were individually housed and maintained on a 12/12-h light/dark cycle at 25 °C and 50–60% humidity. We chose to match the animals by age because of the complications involved in matching by body mass requiring either that the males be severely food restricted or the females much older than the males. Further, complicating matching by body mass, male and female rats have different weight gain trajectories in response to high-fat diet.

Following acclimatization to the facilities, animals were given ad libitum access to water and a custom-made palatable high-fat diet (high-fat diet) that we have used previously [18] (D12451, Research Diets, New Brunswick, NJ, 4.73 kcal/g, 45% butter fat; 19g of butter oil and 1 g of soybean oil to provide essential fatty acids) for 3 weeks prior to surgery and maintained on the diet until the studies were terminated. Animals were assigned to receive either Sham or VSG surgery in a counterbalanced fashion by body weight. Cohort 1 (male, *n* = 6; female, *n* = 6 rats) was studied for hepatic microarray gene expression after surgery while cohort 2 (male, *n* = 20 and female, *n* = 20 rats) was studied for the phenotypic response to surgery.

### Surgical procedures

Four days prior to surgery, body composition was assessed using an EchoMRI analyzer (Houston, TX). Animals were fed Osmolite OneCal liquid diet but no solid-food for 48-h prior to surgery. VSG was performed as previously described [19]. Briefly, it consisted of a midline abdominal laparotomy with exteriorization of the stomach. The lateral 80% of the stomach was excised using an ETS 35-mm staple gun (Ethicon Endo-Surgery, Cincinnati, OH), leaving a tubular gastric remnant in continuity with the esophagus. This gastric sleeve was then reintegrated into the abdominal cavity and the abdominal wall was closed in layers. For the sham surgery, an abdominal laparotomy was performed, light manual pressure was applied with to the exteriorized stomach, and then the abdomen was closed in layers.

For 3 days following surgery, all rats received twice-daily subcutaneous injections of 5 mL saline and 0.20 mL Buprenex® (0.05mg/kg), and animals were maintained on Osmolite liquid diet which was replaced with high fat diet on day 4.

### Microarray studies

During post-operative week 9, animals were fasted for 24 h and then received either 2 mL (males) or 1.3 mL (females) of olive oil. Blood was taken again at 2-h post-gavage and then animals were killed by an injection of Fatal Plus (1 mg/g body weight) and tissues were collected to determine the impact of sex and surgery on liver triglycerides and hepatic gene expression. The time point was chosen based on previously published work that demonstrates it reflects the time point of the initial rise in both plasma and hepatic uptake of olive oil [20]. Olive oil was used as this is the type of fat we used in previous studies [16]. Liver tissue was collected freshly frozen in methyl butane and then stored in −80 °C until further processing. Hepatic RNA was extracted using a QIAGEN miniprep RNA kit (QIAGEN, Inc, Valencia, CA). The microarrays were performed by the CCHMC Genomics Core. The quality of the total RNA was checked by a 2100 Agilent Bioanalyzer using the RNA 6000 Nano Assay. The GeneChip 3’ IVT Express Kit (Affymetrix) was used to make double-stranded cDNA from 0.3 μg of total RNA. An in vitro transcription reaction creates biotin-labeled cRNA target. The cRNA target is chemically fragmented and then hybridized to an Affymetrix Genechip Array. Then, 15 μg of fragmented cRNA was then hybridized to a Rat Genome 230 2.0 Array (Affymetrix). Probe arrays were incubated at 45 °C for 16 h in the hybridization oven 640 (Affymetrix) rotating at 60 rpm. Probe arrays were washed and stained using the Fluidics Station 450 (Affymetrix) utilizing the fluidics protocol FS450-0001. The stain and Antibody solutions are produced by Affymetrix and contained in the Genechip Hybridization Wash and Stain Kit. GeneChips were scanned using the Affymetrix GeneChip Scanner 3000 7G. The .cel and .chp files for the samples were created using the Expression Console software (Affymetrix).

Relative hepatic gene expression from male and female (Sham and VSG treatments) rats was obtained using the Affymetrix Gene Chip Rat 230 2.0. Data was normalized using the RMA algorithm to the median of the control samples (sex-specific Sham). We further filtered the data, requiring a signal intensity of 75 in at least one of the four experimental conditions. In order to generate gene-sets of differentially regulated significant and relevant genes, we formed pairwise comparisons between select conditions with a fold-change cut-off of 1.5 and accepted significance at *p* < 0.05. Additionally, we performed a ranking procedure to select the 500 top- and bottom-expressed genes for each condition. All expression analysis was performed in GeneSpring 12.5. Gene sets were submitted to ToppGene for ontological analysis, which uses unbiased methods to determine gene set enrichment for pathways, biological processes, and molecular functions.

### Body weight, composition, and food intake

In a second cohort of VSG and sham animals, food intake and body weights were measured daily for the first week following surgery. Body composition (fat and lean mass) was determined as described above before high fat diet (3 weeks prior to surgery), 4 d prior to surgery, and then 4 and 16 weeks following surgery. During the postoperative period, several physiological studies were performed to determine the impact of biological sex on physiological responses to bariatric surgery, in particular, aspects of lipid metabolism were evaluated.

### Glucose tolerance and baseline measurements

During postoperative week 5, animals were fasted for ~6 h following the onset of the light. Baseline blood glucose was measured using an AccuChek glucometer. Rats were administered 50% dextrose by oral gavage at a flat dose equivalent to 1.5 g of glucose per the average body mass of the respective sex. Thus, all males received 1.5mL and all females received 0.9mL of 50% dextrose. Blood glucose was then measured at 15, 30, 45, 60, and 120 min following dextrose administration. In addition, plasma from the 0 time point was also used to determine fasting levels of circulating triglyceride and cholesterol.

### Lipid homeostasis

#### Physiologic disappearance of triglycerides

During post-operative week 6, animals were allowed ad libitum access to high fat diet for 24 h prior the experiment. Hoppers were then removed at lights on and tail vein blood sample taken at time 0, 4, 8, and 24 h following hopper removal to assess plasma triglycerides.

#### Lipid absorption through fecal analysis

During post-operative week 8, 24 h fasted rats received a gavage of a lipid emulsion containing 20% soybean oil, 1.2% egg phospholipid, 2.5% glycerin, 2.5% sucrose polybehenate at a volume of 5 mL/kg. Fecal samples were collected 24 and 48 h later. Fecal lipid content was assayed by gas chromatography of fatty acid methyl esters by the UC Mouse Metabolic Phenotyping Core (MMPC). Dietary lipid absorption was estimated using a ratio of total fecal fatty acids to sucrose polybehenate.

#### Post-prandial lipid distribution

During post-operative week 23, animals were fasted for 24h, baseline blood was sampled for subsequent analysis of plasma triglyceride, and then the animals each received a gavage of radioloabeled [9, 10(N)-3H] glycerol trioleate (100 μCi; #NET431L005MC, Perkin Elmer) mixed with 5.0 mL/kg of olive oil. Blood was taken again 2h after the gavage and then animals were sacrificed in a counter-balanced fashion (by sex) by an injection of Fatal Plus (1 mg/g body weight). Tissues were collected to determine the impact of sex and surgery on postprandial lipid distribution, liver triglycerides, and gene expression.

#### Postprandial chylomicron production

During post-operative week 7, animals were fasted for 24 h and then within 1 h of lights received an intraperitoneal injection of 1 g/kg poloxamer 407 (P-407; Sigma-Aldrich, St Louis, MO), a lipoprotein lipase inhibitor. Then 15 min later, a baseline blood sample from the tail vein was collected (*t* = 0) and an intragastric gavage of 0.5 mL/kg olive oil was delivered (average dose: males 240μl and females 130μl olive oil). Tail vein blood was then collected at 2 and 6 h following gavage.

#### Hepatic VLDL production

During the 18th postoperative week, rats were fasted for 24 h, baseline blood samples were collected, and then rats received an intraperitoneal injection of 1 g/kg poloxamer 407 (P-407; Sigma-Aldrich, St Louis, MO). Blood was sampled again at 2, 4, and 6 h after injection.

### Analyses

#### RNA processing and real-time PCR

In order to confirm the findings of the array data performed in cohort 1, liver tissue from the second cohort of animals was collected and hepatic RNA was extracted as described above. cDNA was transcribed using an iScript kit (Bio-Rad Laboratories, Hercules, CA). QPCR was performed using a TaqMan 7900 Sequence Detection System with TaqMan Universal PCR Master Mix and TaqMan Gene Expression Assays (all from Applied Biosystems, Foster City, CA; Primers listed in Table 1).

**Table 1:** Real-time QPCR validation of genes using hepatic samples in cohort 1

Gene Name	Catalog #	Female		Male		Statistics (two-way ANOVA)
		Sham	VSG	Sham	VSG	
*Lipid metabolism*						
ACOX1	Rn01460628_m1	100 ± 7^a^	75 ± 5^b^	91 ± 3	94 ± 7	*p* (surgery × sex) < 0.05
CD36	Rn01442639_m1	100 ± 10^a^	49 ± 6^b^	2 ± 1^c^	4 ± 1^c^	*p* (surgery × sex) < 0.001
CPT1A	Rn00580702_m1	100 ± 9	72 ± 11	90 ± 6	79 ± 12	NS
DGAT2	Rn00584870_m1	100 ± 6^a^	73 ± 6^b^	54 ± 4^b^	53.1 ± 4^b^	*p* (surgery × sex) < 0.05
FASN	Rn00569117_m1	100 ± 12	70 ± 11	17 ± 5	14 ± 2	*p* (sex) < 0.001
LDLR	Rn00598442_m1	100 ± 8^a^	64 ± 5^c^	29 ± 3^b^	31 ± 3^b^	*p* (surgery × sex) < 0.01;
MGAT	Rn00585985_s1	100 ± 4	82 ± 6	68 ± 5	67 ± 6	*p* (sex) < 0.001
PGC1	Rn00590984_m1	100 ± 12	104 ± 12	35 ± 3	68 ± 7	*p* (sex) < 0.001
PPARα	Rn00566193_m1	100 ± 9	59 ± 8	106 ± 10	102 ± 11	*p* (sex) < 0.05, *p* (surgery) < 0.05,
PPARγ	Rn00440945_m1	100 ± 13	67 ± 10	85 ± 13	75 ± 11	*p*(surgery) < 0.05
SREBP	Rn01495769_m1	100 ± 6	68 ± 7	70 ± 10	58 ± 5	*p* (surgery) < 0.05, *p* (sex) < 0.01
*Cholesterol Metabolism*						
ACAT2	Rn01759928_g1	100 ± 11^a^	66 ± 7^b^	20 ± 1^c^	21 ± 3^c^	*p* (surgery × sex) < 0.05;
CYP7a1	Rn00564065_m1	100 ± 24	85 ± 11	40 ± 7	44 ± 15	*p* (sex) < 0.001
MTTP	Rn01522970_m1	100 ± 7^a^	64 ± 5^b^	35 ± 2^c^	37 ± 3^c^	*p* (surgery × sex) < 0.001
LRH1	Rn00572649_m1	100 ± 15^a^	57 ± 5^b^	91 ± 7^a^	92 ± 6^a^	*p* (surgery × sex) < 0.001
*Receptors*						
Erα	Rn00433142_m1	100 ± 6^a^	75 ± 5^b^	81 ± 4^b^	76 ± 5^b^	*p* (surgery × sex) < 0.05
FGFR1	Rn00577234_m1	100 ± 10	106 ± 6	88 ± 5	116 ± 8	*p* (surgery) < 0.05
FXR	Rn00572658_m1	100 ± 7^a^	59 ± 8^b^	74 ± 6^b^	64 ± 5^b^	*p* (surgery × sex) < 0.05
*Gluconeogenesis*						
G6PC	Rn01529640_g1	100 ± 4	80 ± 4	106 ± 6	93 ± 7	P(surgery) < 0.01
PCK1	Rn01529009_g1	100 ± 8	92 ± 12	158 ± 13	185 ± 16	P(sex) < 0.001

Data are presented as mean ± SEM. Groups with different superscript letters are significantly different via Tukey post hoc analysis

#### Tissue and Plasma Analytes

Liver triglycerides were measured using an enzymatic assay (#T7532-120, Pointe Scientific, Canton MI). Plasma was stored at -80 °C until further processing. Plasma was diluted 1:20 in saline in order to measure triglycerides (#TR22421, Infinity Triglyceride Reagent, Thermo Scientific, Waltham, MA). Cholesterol (Infinity Cholesterol, #TR13421, Thermo Scientific, Waltham, MS), total bile acids (#BQ 092A-EALD, BQkits Diagnostics, San Diego, CA), β-hydroxybutyrate (#SBHR-100, Fisher Scientific, Waltham, MA), and non-esterified fatty acids (Wako Diagnostics, Richmond, VA) were measured using enzymatic assays. TGFβ measurements were made using a standard ELISA (#MB100B, R&D Systems, Minneapolis, MN). Estradiol and progesterone assays were performed by the Vanderbilt Hormone Assay and Analytical Services Core (Vanderbilt University, Nashville, TN).

### Statistical analyses

Except for the microarray data which was analyzed as described above, all statistical analyses were performed using GraphPad Prism version 4.0 (GraphPad Software, San Diego, California, USA). To observe time-wise differences, two-way ANOVA (variables: surgery/sex and time) with a Bonferroni post hoc test was used. When time was not a variable, a two-way ANOVA for sex and surgery with a Bonferroni post hoc test was used. All results are given as means ± SEM. Results were considered statistically significant when *p* < 0.05.

## Results

**Fig. 1:**
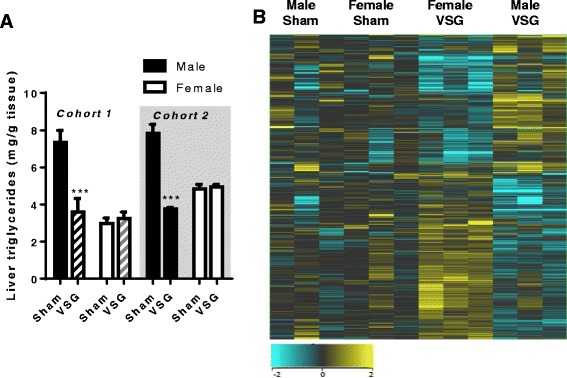
**a** Hepatic triglyceride content in the two cohorts of rats studied. Cohort 1 (*n* = 3/grp), Cohort 2 (*n* = 8–10/grp) **b**. Heat map of hepatic gene expression in Sham male vs. female and VSG male vs. female rats as quantified by Affymetrix array (*n* = 3/group). ***p < 0.001. Data are presented as mean ± SEM

Previous literature suggests that VSG reduces hepatic triglycerides in male mice [17, 21] but not in previously pregnant female rats [8]. Thus, we generated a small cohort of virgin male and female rats in order to do a side by side comparison of the hepatic lipid response to Sham vs. VSG surgery. We found that Sham males had greater hepatic triglycerides than Sham females (Fig. 1a) and after VSG, males had a significant reduction in hepatic triglycerides (student’s t test, *p* < 0.001). Conversely, females had no surgery-induced improvements in hepatic triglycerides (Fig. 1a). This phenomenon was recapitulated in a second cohort of animals (Fig. 1a).

**Fig. 2:**
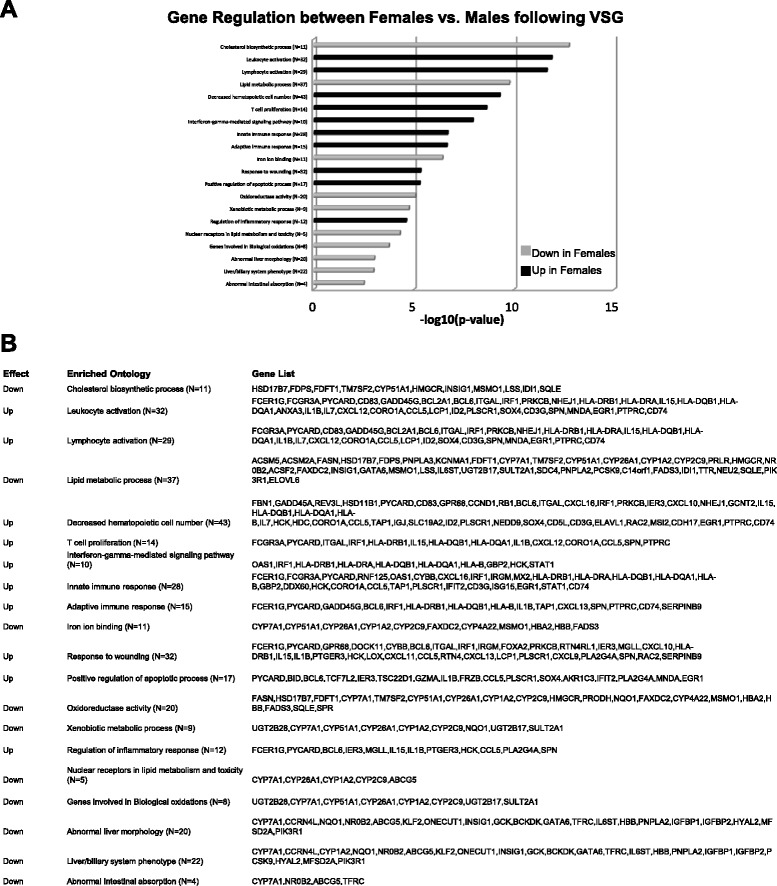
**a** Hepatic gene changes in males vs. females following VSG. *Gray bars* designate categories that were down-regulated in VSG females vs. VSG males. Black bars designate genes that were up-regulated in VSG females vs. VSG males. **b** Gene lists for categories of gene changes exhibited in panel **a** (*n* = 3/group)

We also utilized a gene array analysis to determine if patterns of hepatic gene expression changes after VSG would explain this sex difference. Previous work using mRNA-seq analysis of male livers after VSG has found increased expression of genes related to bile acid metabolism, and fatty acid oxidation, and decreased expression of genes related to lipogenic pathways [17]. However, in our females we found that VSG downregulated lipid, glucose, and bile acid metabolic pathway genes (Additional file 1: Figure S1). While Fig. 1b shows many similarities in gene expression between obese Sham males and females, in response to VSG, males and females demonstrated directly opposing gene-related changes as clearly visualized in the heat map (Fig. 1b). Specifically, genes that regulated lipid metabolism (cholesterol biosynthesis, lipid metabolism, the hepatic biliary system, nuclear receptors in lipid metabolism) were all down-regulated by VSG in females (Fig. 2a, b). Other VSG-induced gene changes in females were increases in genes related to immune activation (i.e., leukocyte activation, lymphocyte activation, hematopoietic number, T cell proliferation, innate immune response, adaptive immune response, regulation of inflammatory response) (Fig. 2a, b). These latter findings will be probed in future studies.

We then performed qPCR on liver tissue in order to validate the striking microarray findings. Similar to the array and consistent with our in vivo findings, genes that regulate or are involved in cholesterol synthesis and VLDL production (MTTP, DGAT2, ACAT2, CD36, LDLR, LRH1) and lipid metabolism (ACOX1, SREBP, ERα) were down-regulated by VSG in females (Table 1).

**Fig. 3:**
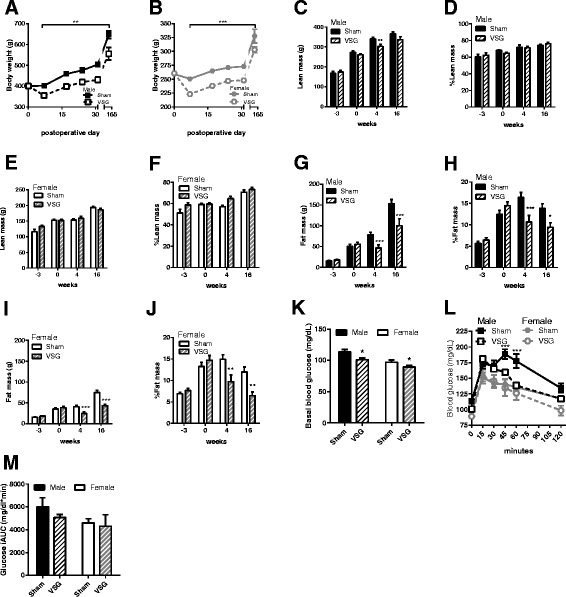
Parallel comparison of male (*n* = 9–10) and female (*n* = 9–10) body weight, body composition and glucose tolerance after vertical sleeve gastrectomy (VSG) and Sham-VSG (Sham) surgeries. **a** Body mass was significantly lower from postoperative day 6 to day 165 **b**. Body mass was significantly lower from postoperative day 6 to day 165 **c**. Male lean body mass was significantly lower 4 wks but not at 16 wks postoperatively in VSG vs. Sham groups. **d** % lean mass in males was not significantly different at any time point in VSG vs. sham groups. **e** & **f** Female absolute and relative (%) lean body mass was not significantly different between groups at any time point. **g**, **h** Male absolute (g) and relative (%) fat mass were significantly lower in VSG vs. Sham at 4 and 16wks postoperatively. **i**, **j** Female absolute and relative (%) fat mass were significantly lower in VSG vs. Sham at 4 and 16wks postoperatively. **k** Basal blood glucose after 6 h fasting was significantly lower in VSG vs. Sham in both males and females. **l** Blood glucose levels during an oral glucose tolerance test were significantly lower in VSG vs. sham in males only. **m** Integrated glucose area under the curver during the OGTT in H. *p < 0.05, **p < 0.01, ***p < 0.001. Data are presented as mean ± SEM

Because of the clear differences in both hepatic triglycerides and gene expression, we generated a second cohort of animals in order determine differences in the physiological regulation of lipid metabolism. We found that both male and female rats lost a significant amount of body weight in the first 31 days after VSG and remained at a reduced body weight until they were terminated at the end of the study (main effect of surgery, *p* < 0.001 and time *p* < 0.001) (Fig. 3a, b). Following VSG, males exhibited a transient reduction in lean body mass at 4 weeks postoperatively (student’s t test, *p* < 0.01) (Fig. 3c) that was no longer significant at week 16. For females, there was no significant difference in lean body mass at any time point between surgical groups (Fig. 3d). Both males and females had a significantly lower body fat mass at 4 and 16 weeks after VSG vs. sham surgeries (*p* < 0.001) (Fig. 3e, f). The absolute change in body fat mass was less in females as compared to males likely due to the comparatively smaller pre-surgical fat mass. During postoperative week 5, both male and female VSG animals had significantly reduced fasting blood glucose (main effect of surgery, *p* < 0.01) (Fig. 3g) and females overall had reduced glucose levels in comparison to males (main effect of sex, *p* < 0.001) (Fig. 3g). After VSG, males had significantly reduced glucose levels 45 and 60 min after glucose gavage compared to Sham animals (surgery × time, *p* < 0.001) (Fig. 3h). Glucose response to the oral glucose gavage was not significantly different between sham and VSG surgeries at any time point in the females.

We measured baseline characteristics of several plasma metabolites and hormones and these reflected the expected sex and surgery-induced differences (Table 2). Specifically, males had significantly greater non-esterified fatty acids (NEFA; *p* < 0.001) but lower cholesterol (*p* < 0.001), bile acids, and estradiol levels compared to females (*p* < 0.001). In both sexes, surgery reduced plasma triglycerides (*p* < 0.05), plasma NEFA (*p* < 0.01), fat absorption (*p* < 0.01), plasma estradiol levels (*p* < 0.01), and significantly increased total plasma bile acid levels (*p* < 0.05).

**Table 2:** Plasma metabolite measurements *p < 0.05, **p < 0.01, ***p < 0.001. Data are presented as mean ± SEM

Metabolite	Female		Male		2-Way ANOVA Statistics
	Sham	VSG	Sham	VSG	
Triglycerides (mg/dl)	339 ± 44	107 ± 21	439 ± 55	153 ± 27	*p* (surgery) < 0.05
Cholesterol (mg/dl)	91 ± 4	87 ± 3	71 ± 4	62 ± 5	*p* (sex) < 0.001
β-Hydroxybutyrate (ng/ml)	1.5 ± 0.1	1.5 ± 0.1	1.9 ± 0.1	1.7 ± 0.2	NS
NEFA (mEq/L)	1.8 ± 0.1	1.6 ± 0.1	2.3 ± 0.1	1.9 ± 0.1	*p* (sex) < 0.001; *p* (surgery) < 0.01
Total bile acids (μM/L)	23 ± 3	93 ± 19	13 ± 5	45 ± 9	*p* (sex) < 0.001; *p* (surgery) < 0.05
Fat absorption (%)	93 ± 2	82 ± 5	90 ± 4	82 ± 3	*p* (surgery) < 0.01
Estradiol (pg/ml)	70 ± 11	39 ± 4	28 ± 2	20 ± 3	*p* (sex) < 0.001; *p* (surgery) < 0.01
Progesterone (ng/ml)	6.4 ± 1.5	4.9 ± 0.6	ND	ND	NS

**Fig. 4:**
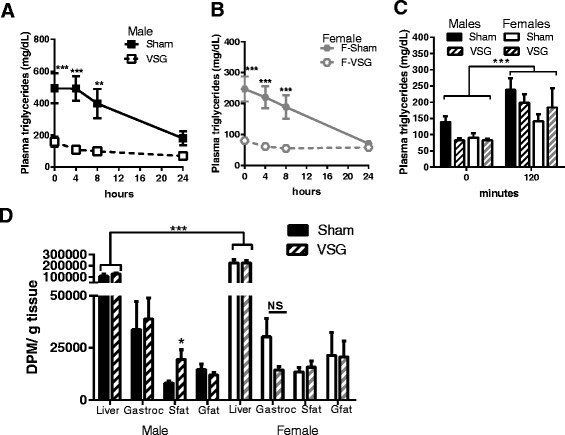
Postprandial lipid uptake. Plasma triglyceride levels in ad libitum fed rats (*t* = 0) and following 4, 8, and 24 h of fasting in **a** males and **b** females. **c** Plasma triglycerides in males and females following the ^3^H glycerol trioleate + olive oil gavage. **d** Plasma ^3^H in male rats following a 100μCi + 5ml/kg olive oil gavage. **e** Tissue ^3^H content 2h after the gavage in the male and female liver, gastrocnemius (Gastroc), subcutaneous fat (Sfat), and gonadal fat (Gfat). (n = 9-10 per group). *p < 0.05, **p < 0.01, ***p < 0.001. Data are presented as mean ± SEM

We next performed a series of studies to examine if organ-specific lipid handling was differentiated by biological sex and could explain the lack of reduction in hepatic triglycerides. We found that both male and female VSG animals had significantly reduced plasma triglycerides in an ad lib fed state and at 4, and 8 h of fasting, compared to the Sham animals (surgery × time interaction, *p* < 0.0001) (Fig. 4a, b). By 24-h of fasting, all groups had similar levels of triglycerides.

We then examined whether postprandial lipids were preferentially trafficked to the liver in VSG females compared to males. Basal levels of plasma triglycerides were greatest in sham surgery males compared to all other groups (Fig. 4c; *p* < 0.01) and following the gavage of a radiolabeled lipid emulsion lead to there were similar increases in plasma triglycerides (Fig. 4c) in the in males and females regardless of surgery. In the liver, and independent of surgery, females had significantly greater ^3^H uptake than males (main effect of sex, *p* < 0.001; Fig. 4e). However, surgery did not alter the amount of ^3^H-glycerol trioleate uptake into the liver, gastrocnemius, or gonadal fat (epididymal for male and peri-ovarian for females) tissues but did cause a significant increase of ^3^H-glycerol trioleate in subcutaneous fat of VSG compared to Sham males (*p* < 0.05) (Fig. 4e).

**Fig. 5:**
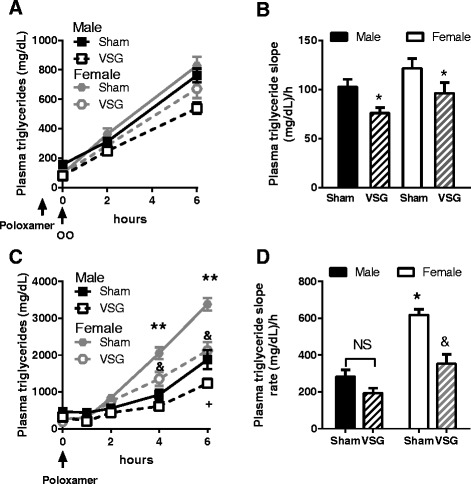
Intestinal and hepatic contribution to plasma triglycerides. **a** Postprandial chylomicron production as indexed by the plasma triglyceride response to poloxomer 407 in fed rats. **b** Slope of the rise in triglycerides in **a** (n = 8-10/grp). **c** Hepatic VLDL production as indexed by plasma triglyceride response to poloxamer 407 in fasted rats. **d** Slope of triglyceride appearance based on the results in **c**. *p < 0.05 VSG vs. sham; **p < 0.05 sham females vs. all other groups; +*p* < 0.05 VSG male vs. Sham male; &VSG female vs. VSG male. Data are presented as mean ± SEM

To determine if surgery impacted intestinal chylomicron production in a sex-dependent manner, we administered poloxamer 407, a drug that prevents plasma triglyceride hydrolysis, to fed animals. Under these conditions, the increase in plasma triglycerides is a surrogate measure for chylomicron production. We no impact of sex, per se, but found that male and female VSG animals had similarly increased triglyceride levels and thus reduced rates of triglyceride appearance compared to sham surgery animals (main effect of surgery; Fig. 5a, b). Thus, postprandial chylomicron production was reduced after VSG in both males and females.

We next determined whether hepatic VLDL production, which is the predominant source of triglycerides under fasting conditions, was altered by biological sex. To do this, we administered poloxamer 407 to 16h fasted animals. Sham females had significantly greater VLDL production compared to all other groups and importantly, VSG lowered this to the level of males at 4 and 6 h (Fig. 5c). Likewise, the rate of triglyceride appearance over time (slope of the data in Fig. 5d) was significantly reduced by VSG in females but not males (sex × surgery interaction, *p* < 0.001). These data suggest that hepatic triglyceride export is slowed by VSG in females.

## Discussion

Men and women have distinct regulation of lipid metabolism. These differences go beyond region-specific fat distribution. For example, at the same level of obesity, women have a lower risk for type 2 diabetes mellitus (T2DM) and cardiovascular disease [22], in response to metabolic stress, such as weight loss or exercise, fat is mobilized more readily in women [11–14], and lastly genome-wide association studies have identified multiple sex-dependent loci in medically-relevant traits [23]. Here, we find that bariatric surgery emphasizes sex differences specifically in hepatic lipid handling such that genes that regulate lipid metabolism are down-regulated by VSG resulting in lower VLDL export and maintenance of sham-level hepatic triglycerides. This is in contrast to the surgery-induced reduction in hepatic triglycerides by VSG in males which is not explained by changes in lipid uptake or export and likely is reflected by intrahepatic changes in metabolism [21].

Similar to clinical reports, VSG was a successful strategy in causing weight loss and improving plasma glucose and lipid levels in both male and female rats [24–26]. It is only to this extent of physiology that most clinical studies have probed and they report similar responses to surgery between men and women. While some studies have observed similar clinical responses to bariatric surgery between men and women [27], exploration of the bariatric outcomes longitudinal database (BOLD) did find that sex contributed to the variability of surgical outcome [28]. However, a true physiological comparison of the responses to surgery in men vs. women is limited by the small number of male patients in addition to the need for the appropriate resources for these studies. For example, hormone status likely influences the surgical outcome; however, the large- and even small-scale studies that included sex as a variable in models to evaluate surgical success were unable to actually measure hormone status [9, 28].

Both clinical and preclinical data clearly reflect sexual dimorphisms in lipoprotein profiles and indicate that females rely more on lipid flux during times of metabolic stress [13, 14, 29]. Importantly, our rodent model results present here also have parallels to clinical work. As we observed with our rats, women have greater VLDL production than men [30] and this has been found to be due specifically to greater levels of estradiol in women [31]. Lastly, in a group of eight women and one man, VLDL production was found to be reduced by RYGB [31]. Whether this would influence hepatic triglyceride stores in patients remains to be determined. Retrospective studies suggest an improvement in hepatic lipids after RYGB [31–34]. However, a few studies report either a lack of improvement or even increased steatosis after RYGB [35, 36]. The bottom line is that even though there is an average improvement of liver fat, this does not happen in all of patients after RYGB suggesting that there is individual variation in response to the surgery. In addition, postoperative timing of hepatic lipid measures [37], type of surgery [34] and technique used to assess hepatic lipids could all influence conclusions about the impact of surgery on hepatic lipids.

The present data support previous findings that hepatic lipid flux is quite different in males and females; differences which typically protect the female from hepatic lipid accumulation. However, VSG changes this metabolic profile preventing further reductions in hepatic triglycerides in female rodents. It is possible that the lack of an effect of VSG on hepatic triglycerides in females is due to a floor effect; i.e., the Sham-operated females already had lower hepatic triglyceride levels compared to males. Our previous research demonstrated that high fat vs. chow-fed female rats have increased hepatic triglycerides [8]. Thus, in our current study we should have enough of an experimental signal to detect a surgery-induced reduction in hepatic triglycerides if one existed. It is important to note that this level of hepatic triglycerides is still lower than Sham males, and explains why glucose tolerance was lower in Sham and VSG females vs. Sham males. Interestingly, our previous research has shown that VSG prior to pregnancy leads to a long-term increase in hepatic triglycerides but these animals maintained a surgery-induced improvement in glucose tolerance [8]. Women, and female zucker diabetic fatty rats, ob/ob and db/db mice all display protection from diabetes or hyperglycemia, respectively compared to men/males [22, 38–41]. While we hypothesize that VSG is yet another model whereby females are able to protect hepatic glucose metabolism in the face of higher liver triglycerides, we also admit that the short length of time on HFD, as well as the fact that the animals are maintained on HFD post-operatively, may limit the translation of this work to humans with longer-standing obesity and post-operative changes in feeding behavior that are metabolically favorable.

An interaction between reproductive hormones and surgery likely drives changes in lipid metabolism in females. Estradiol has been demonstrated to be a primary mechanism driving sex differences in lipid metabolism [13, 42]. In an elegant series of studies, Zhu et al. [42] found that hepatic estrogen receptor alpha (ER α) signaling plays a crucial role in regulating lipid flux across the liver. Namely, hepatic ER α signaling limited liver fat synthesis but maintained triglyceride export in the setting of hyperinsulinemia with the net result of reduced hepatic triglycerides. Thus, our finding that hepatic triglycerides failed to reduce after VSG may be explained by the reduced hepatic ERα expression (Table 1) also observed in the females after VSG. Interestingly, reduced hepatic ER α has been found in patients with non-alcoholic steatohepatitis [43] demonstrating clinical relevance of hepatic estrogen signaling.

## Conclusions

In conclusion, our results indicate that male and female rodents have similar qualitative responses to VSG. However, there are large-scale changes in the genes regulating lipid metabolism in female but not male rodents in response to VSG. In addition, VSG causes less export of triglycerides in a fasting state in females while lipid uptake and export are not changed by VSG in males. As a result, while males have a significant reduction females retain high-fat levels hepatic triglycerides. In our efforts to understand the molecular underpinnings of bariatric surgery, research has mostly neglected the contribution of sex to outcomes. The current results demonstrate that studying female rodents is necessary to advance our understanding of the molecular mechanisms of bariatric surgery for the greater than 80% of bariatric surgery patients that are female.

## Additional file

Additional file 1: Figure S1. Hepatic gene changes in females following VSG and sham surgeries. Gray bars designate categories that were down-regulated in VSG. Black bars designate genes that were up-regulated in VSG. B. Gene lists for categories of gene changes exhibited in panel A (*n* = 3/group). (PDF 112 kb)

## Abbreviations

NEFA: Non-esterified fatty acids
qPCR: Quantitative polymerase chain reaction
RYGB: Roux-en-Y gastric bypass
T2DM: Type 2 diabetes mellitus
VLDL: Very low density lipoprotein
VSG: Vertical sleeve gastrectomy

## References

[1] Karamanakos SN, Vagenas K, Kalfarentzos F, Alexandrides TK (2008). Weight loss, appetite suppression, and changes in fasting and postprandial ghrelin and peptide-YY levels after Roux-en-Y gastric bypass and sleeve gastrectomy: a prospective, double blind study. Ann Surg.

[2] Gluck B, Movitz B, Jansma S, Gluck J, Laskowski K (2011). Laparoscopic Sleeve Gastrectomy is a Safe and Effective Bariatric Procedure for the Lower BMI (35.0-43.0 kg/m(2)) Population. Obes Surg.

[3] Abu-Jaish W, Rosenthal RJ (2010). Sleeve gastrectomy: a new surgical approach for morbid obesity. Expert Rev Gastroenterol Hepatol.

[4] Hao Z, Townsend RL, Mumphrey MB, Patterson LM, Ye J, Berthoud H-R. Vagal Innervation of Intestine Contributes to Weight Loss After Roux-en-Y Gastric Bypass Surgery in Rats. Obes Surg. 2014. 10.1007/s11695-014-1338-3 PMC4224982 24972684

[5] Hatoum IJ, Stylopoulos N, Vanhoose AM, Boyd KL, Yin DP, Ellacott KLJ (2012). Melanocortin-4 receptor signaling is required for weight loss after gastric bypass surgery. J Clin Endocrinol Metab.

[6] Liou AP, Paziuk M, Luevano J-M, Machineni S, Turnbaugh PJ, Kaplan LM (2013). Conserved shifts in the gut microbiota due to gastric bypass reduce host weight and adiposity. Sci Transl Med.

[7] Mokadem M, Zechner JF, Margolskee RF, Drucker DJ, Aguirre V (2014). Effects of Roux-en-Y gastric bypass on energy and glucose homeostasis are preserved in two mouse models of functional glucagon-like peptide-1 deficiency. Mol Metab.

[8] Grayson BE, Schneider KM, Woods SC, Seeley RJ (2013). Improved rodent maternal metabolism but reduced intrauterine growth after vertical sleeve gastrectomy. Sci Transl Med.

[9] Ochner CN, Teixeira J, Geary N, Asarian L (2013). Greater short-term weight loss in women 20-45 versus 55-65 years of age following bariatric surgery. Obes Surg.

[10] Asarian L, Abegg K, Geary N, Schiesser M, Lutz TA, Bueter M (2012). Estradiol increases body weight loss and gut-peptide satiation after Roux-en-Y gastric bypass in ovariectomized rats. Gastroenterology.

[11] Perreault L, Lavely JM, Kittelson JM, Horton TJ (2004). Gender differences in lipoprotein lipase activity after acute exercise. Obes Res.

[12] Perreault L, Lavely JM, Bergman BC, Horton TJ (2004). Gender differences in insulin action after a single bout of exercise. J Appl Physiol.

[13] Sandoval DA, Ertl AC, Richardson MA, Tate DB, Davis SN (2003). Estrogen blunts neuroendocrine and metabolic responses to hypoglycemia. Diabetes.

[14] Horton TJ, Pagliassotti MJ, Hobbs K, Hill JO (1998). Fuel metabolism in men and women during and after long-duration exercise. J Appl Physiol.

[15] Zhang Y, Klein K, Sugathan A, Nassery N, Dombkowski A, Zanger UM (2011). Transcriptional profiling of human liver identifies sex-biased genes associated with polygenic dyslipidemia and coronary artery disease. PLoS One.

[16] Stefater MA, Sandoval DA, Chambers AP, Wilson-Perez HE, Hofmann SM, Jandacek R (2011). Sleeve gastrectomy in rats improves postprandial lipid clearance by reducing intestinal triglyceride secretion. Gastroenterology.

[17] Myronovych A, Kirby M, Ryan KK, Zhang W, Jha P, Setchell KD (2014). Vertical sleeve gastrectomy reduces hepatic steatosis while increasing serum bile acids in a weight-loss-independent manner. Obesity (Silver Spring).

[18] Woods SC, Seeley RJ, Rushing PA, D’Alessio DA, Tso P (2003). A controlled high-fat diet induces an obese syndrome in rats. J Nutr.

[19] Stefater M, Pérez-Tilve D, Chambers AP, Wilson-Pérez HE, Sandoval D, Berger J (2010). Sleeve gastrectomy induces loss of weight and fat mass in obese rats, but does not affect leptin sensitivity. Gastroenterology.

[20] Jackman MR, Kramer RE, MacLean PS, Bessesen DH (2006). Trafficking of dietary fat in obesity-prone and obesity-resistant rats. Am J Physiol Endocrinol Metab.

[21] Myronovych A, Salazar-Gonzalez R-M, Ryan KK, Miles L, Zhang W, Jha P (2014). The role of small heterodimer partner in nonalcoholic fatty liver disease improvement after sleeve gastrectomy in mice. Obesity.

[22] Karastergiou K, Smith SR, Greenberg AS, Fried SK (2012). Sex differences in human adipose tissues - the biology of pear shape. Biol Sex Differ.

[23] Gilks WP, Abbott JK, Morrow EH (2014). Sex differences in disease genetics: evidence, evolution, and detection. Trends Genet.

[24] Rosenthal R, Li X, Samuel S, Martinez P, Zheng C (2009). Effect of sleeve gastrectomy on patients with diabetes mellitus. Surg Obes Relat Dis.

[25] Rubino F, Gagner M, Gentileschi P, Kini S, Fukuyama S, Feng J (2004). The Early Effect of the Roux-en-Y Gastric Bypass on Hormones Involved in Body Weight Regulation and Glucose Metabolism. Ann Surg.

[26] Peterli R, Wolnerhanssen B, Peters T, Devaux N, Kern B, Christoffel-Courtin C (2009). Improvement in glucose metabolism after bariatric surgery: comparison of laparoscopic Roux-en-Y gastric bypass and laparoscopic sleeve gastrectomy: a prospective randomized trial. Ann Surg.

[27] Kennedy-Dalby A, Adam S, Ammori BJ, Syed AA (2014). Weight loss and metabolic outcomes of bariatric surgery in men versus women - A matched comparative observational cohort study. Eur J Intern Med.

[28] Benoit SC, Hunter TD, Francis DM, De La Cruz-Munoz N. Use of Bariatric Outcomes Longitudinal Database (BOLD) to Study Variability in Patient Success After Bariatric Surgery. Obes Surg. 2014. 10.1007/s11695-014-1197-y 24570089

[29] Mittendorfer B, Horowitz JF, Klein S (2001). Gender differences in lipid and glucose kinetics during short-term fasting. Am J Physiol Endocrinol Metab.

[30] Magkos F, Patterson BW, Mohammed BS, Klein S, Mittendorfer B (2007). Women produce fewer but triglyceride-richer very low-density lipoproteins than men. J Clin Endocrinol Metab.

[31] Klein S, Mittendorfer B, Eagon JC, Patterson B, Grant L, Feirt N (2006). Gastric bypass surgery improves metabolic and hepatic abnormalities associated with nonalcoholic fatty liver disease. Gastroenterology.

[32] Barker KB, Palekar NA, Bowers SP, Goldberg JE, Pulcini JP, Harrison SA (2006). Non-alcoholic steatohepatitis: effect of Roux-en-Y gastric bypass surgery. Am J Gastroenterol.

[33] Clark JM, Alkhuraishi ARA, Solga SF, Alli P, Diehl AM, Magnuson TH (2005). Roux-en-Y gastric bypass improves liver histology in patients with non-alcoholic fatty liver disease. Obes Res.

[34] Mattar SG, Velcu LM, Rabinovitz M, Demetris AJ, Krasinskas AM, Barinas-Mitchell E (2005). Surgically-induced weight loss significantly improves nonalcoholic fatty liver disease and the metabolic syndrome. Ann Surg.

[35] Csendes A, Smok G, Burgos AM (2006). Histological findings in the liver before and after gastric bypass. Obes Surg.

[36] Mottin CC, Moretto M, Padoin AV, Kupski C, Swarowsky AM, Glock L (2005). Histological behavior of hepatic steatosis in morbidly obese patients after weight loss induced by bariatric surgery. Obes Surg..

[37] Johansson L, Roos M, Kullberg J, Weis J, Ahlström H, Sundbom M (2008). Lipid mobilization following Roux-en-Y gastric bypass examined by magnetic resonance imaging and spectroscopy. Obes Surg.

[38] Horton TJ, Gayles EC, Prach PA, Koppenhafer TA, Pagliassotti MJ (1997). Female rats do not develop sucrose-induced insulin resistance. Am J Physiol.

[39] D’souza AM, Asadi A, Johnson JD, Covey SD, Kieffer TJ (2014). Leptin deficiency in rats results in hyperinsulinemia and impaired glucose homeostasis. Endocrinology.

[40] Hevener A, Reichart D, Janez A, Olefsky J (2002). Female rats do not exhibit free fatty acid-induced insulin resistance. Diabetes.

[41] Weigt C, Hertrampf T, Flenker U, Hülsemann F, Kurnaz P, Fritzemeier KH (2015). Effects of estradiol, estrogen receptor subtype-selective agonists and genistein on glucose metabolism in leptin resistant female Zucker diabetic fatty (ZDF) rats. J Steroid Biochem Mol Biol.

[42] Zhu L, Brown WC, Cai Q, Krust A, Chambon P, McGuinness OP, et al. Estrogen treatment after ovariectomy protects against fatty liver and may improve pathway-selective insulin resistance. Diabetes. 2012;Epub Sept:1–11. 10.2337/db11-1718 PMC3554377 22966069

[43] Erkan G, Yilmaz G, Konca Degertekin C, Akyol G, Ozenirler S (2013). Presence and extent of estrogen receptor-alpha expression in patients with simple steatosis and NASH. Pathol Res Pract.

