# Supplementary material for: Bariatric surgery emphasizes biological sex differences in rodent hepatic lipid handling
Source: Biol Sex Differ. 2017 Jan 28;8:4. doi: 10.1186/s13293-017-0126-x (PMC5273842; doi:10.1186/s13293-017-0126-x)
Supplement: Additional file 1: Figure S1. — Hepatic gene changes in females following VSG and sham surgeries. Gray bars designate categories that were down-regulated in VSG. Black bars designate genes that were up-regulated in VSG. B. Gene lists for categories of gene changes exhibited in panel A (n = 3/group). (PDF 112 kb) [file 13293_2017_126_MOESM1_ESM.pdf]

# Figure S1

**A**

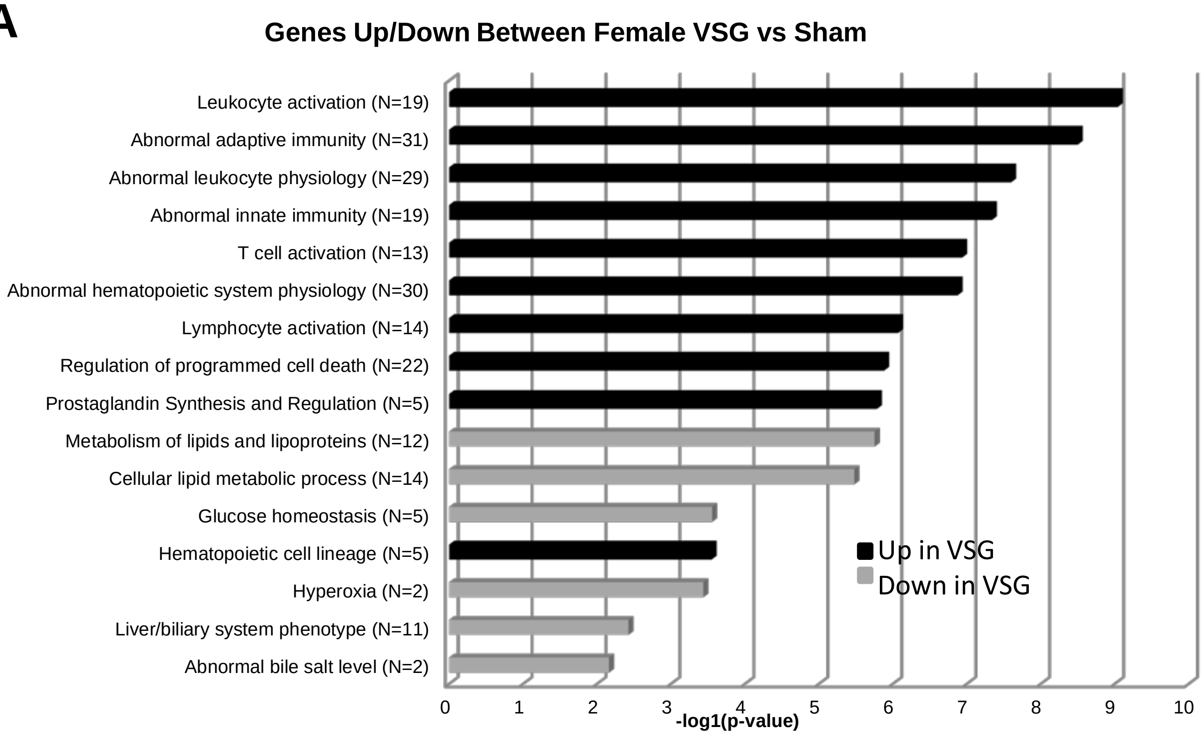


**B**

| Effect | Enriched Ontology | Gene List |
|---|---|---|
| Up | Leukocyte activation (N=19) | FCER1G,ANXA3,IL1B,IL7,TLR1,FCGR3A,GADD45G,IGFBP2,BCL2A1,FAS,PLSCR1,SOX4,CD300A,PRKCB,AIF1,EGR1,PTPRC,NHEJ1,CD74 |
| Up | Abnormal adaptive immunity (N=31) | FCER1G,GZMA,SLPI,IL1B,PTGER3,IL7,TLR1,GADD45G,CYBB,PLAC8,BCL2A1,TLR8,FAS,IGJ,ABCG1,NEDD9,CD300A,PLA2G4A,EMR1,RAC2,PRKCB,ITGA6,MST1,FADS2,CSF1R,EGR1,PTPRC,NHEJ1,LY86,CD74,GBP2 |
| Up | Abnormal leukocyte physiology (N=29) | FCER1G,GZMA,SLPI,IL1B,IL7,TLR1,GADD45G,CYBB,PLAC8,BCL2A1,TLR8,FAS,IGJ,ABCG1,NEDD9,CD300A,PLA2G4A,EMR1,RAC2,PRKCB,ITGA6,MST1,FADS2,EGR1,PTPRC,NHEJ1,LY86,CD74,GBP2 |
| Up | Abnormal innate immunity (N=19) | FCER1G,SLPI,IL1B,TLR1,GADD45G,CYBB,PLAC8,BCL2A1,FAS,IGJ,ABCG1,PLA2G4A,EMR1,RAC2,MST1,FADS2,EGR1,PTPRC,GBP2 |
| Up | T cell activation (N=13) | IL1B,IL7,FCGR3A,GADD45G,IGFBP2,BCL2A1,FAS,SOX4,AIF1,EGR1,PTPRC,NHEJ1,CD74 |
| Up | Abnormal hematopoietic system physiology (N=30) | FCER1G,GZMA,SLPI,IL1B,PTGS1,IL7,TLR1,GADD45G,CYBB,PLAC8,BCL2A1,TLR8,FAS,IGJ,ABCG1,NEDD9,CD300A,PLA2G4A,EMR1,RAC2,PRKCB,ITGA6,MST1,FADS2,EGR1,PTPRC,NHEJ1,LY86,CD74,GBP2 |
| Up | Lymphocyte activation (N=14) | IL1B,IL7,FCGR3A,GADD45G,IGFBP2,BCL2A1,FAS,SOX4,PRKCB,AIF1,EGR1,PTPRC,NHEJ1,CD74 |
| Up | Regulation of programmed cell death (N=22) | FCER1G,GZMA,DAP,IL1B,HSPB1,IL7,FRZB,PDK4,PLAC8,BCL2A1,RTN4,FAS,PLSCR1,SOX4,PLA2G4A,ITGA6,AIF1,BEX2,EGR1,PTPRC,PPP2R2B,CD74 |
| Up | Prostaglandin Synthesis and Regulation (N=5) | HSD11B1,ANXA3,PTGS1,PTGER3,PLA2G4A |
| Down | Metabolism of lipids and lipoproteins (N=12) | HMGCR,FASN,IDI1,ABCG5,PTGIS,PNPLA3,SQLE,CYP7A1,MVD,PIK3R1,ELOVL6,FADS3 |
| Down | Cellular lipid metabolic process (N=14) | ACSM2A,HMGCR,FASN,IDI1,HAO2,PTGIS,PNPLA3,CYP7A1,MVD,PIK3R1,PCSK9,ELOVL6,GATA6,FADS3 |
| Down | Glucose homeostasis (N=5) | ACSM2A,HMGCR,CYP7A1,GCK,SYBU |
| Up | Hematopoietic cell lineage (N=5) | IL1B,IL7,ITGA6,CSF1R,TFRC |
| Down | Hyperoxia (N=2) | HBB,HBA1 |
| Down | Liver/biliary system phenotype (N=11) | ABCG5,MFSD2A,CYP7A1,HBB,PIK3R1,CCRN4L,GCK,PCSK9,BCKDK,GATA6,BCL6 |
| Down | Abnormal bile salt level (N=2) | CYP7A1,PCSK9 |
